# The combined use of 2D scout and 3D axial CT images to accurately determine the catheter tips for high‐dose‐rate brachytherapy plans

**DOI:** 10.1002/acm2.13184

**Published:** 2021-02-27

**Authors:** Kun Qing, Ning J. Yue, Lara Hathout, Chi Ma, Meral Reyhan, Jiahua Zhu, Ke Nie, Gilbert Monte, Irina Vergalasova

**Affiliations:** ^1^ Department of Radiation Oncology Cancer Institute of New Jersey, Rutgers University New Brunswick NJ 08901 USA; ^2^ Department of Radiation Oncology City of Hope National Medical Center Duarte CA 91010 USA

**Keywords:** brachytherapy, HDR, catheter localization, CT, scout

## Abstract

**Purpose:**

To develop a method combining CT scout images with axial images to improve the localization accuracy of catheter tips in high‐dose‐rate (HDR) brachytherapy treatments.

**Materials and Methods:**

CT scout images were utilized along with conventionally reconstructed axial images to aid the localization of catheter tips used during HDR treatment planning. A method was developed to take advantage of the finer image resolution of the scout images to more precisely identify the tip coordinates. The accuracies of this method were compared with the conventional method based on the axial CT images alone, for various slice thicknesses, in a computed tomography dose index (CTDI) head phantom. A clinical case which involved multiple interstitial catheters was also selected for the evaluation of this method. Locations of the catheter tips were reconstructed with the conventional CT‐based method and this newly developed method, respectively. Location coordinates obtained via both methods were quantitatively compared.

**Results:**

Combination of the scout and axial CT images improved the accuracy of identification and reconstruction of catheter tips along the longitudinal direction (i.e., head‐to‐foot direction, more or less parallel to the catheter tracks), compared to relying on the axial CT images alone. The degree of improvement was dependent on CT slice thickness. For the clinical patient case, the coordinate differences of the reconstructed catheter tips were 2.6 mm ± 0.9 mm in the head‐to‐foot direction, 0.4 mm ± 0.2 mm in the left‐to‐right direction, and 0.6 mm ± 0.2 mm in the anterior‐to‐posterior direction, respectively.

**Conclusion:**

Combining CT scout and axial images demonstrates the ability to provide a more accurate identification and reconstruction of the interstitial catheter tips for HDR brachytherapy treatment, especially in the longitudinal direction. The method developed in this work has the potential to be implemented clinically together with automatic segmentation in modern brachytherapy treatment planning systems, in order to improve the reconstruction accuracy of HDR catheters.

## 1 INTRODUCTION

Brachytherapy is a radiation treatment modality of choice to deliver relatively high doses to a local volume of interest with sharp dose fall‐off beyond the treatment region. Due to this inherent advantage, brachytherapy, especially high‐dose‐rate (HDR) brachytherapy, has become an important component for curative management of cancers such as cervical and prostate cancer.^1^, ^2^ For a typical CT‐based HDR treatment, scout images of the patient are initially acquired for the proper selection of the field‐of–view (FOV) and to ensure that the FOV properly includes the appropriate anatomical region‐of‐interest (ROI). The scout is then followed by a multi‐slice CT scan (MSCT) covering the ROIs for digitization, contouring and planning purposes. During the treatment planning process, identification of the implanted catheters needs to be accurately and precisely localized and reconstructed to ensure the accuracy of the HDR source positions and the calculated dose distribution. Inaccurate localization of the catheters, especially their tips, which can often be difficult to identify on images, directly affects the determination of dwell positions of HDR source in the plan, which subsequently influences the accuracy of dose delivery and thus may potentially lead to negative impacts on the treatment outcomes.^3^ Currently, identification of the implanted catheters in the scope of patient anatomy primarily relies on the MSCT images.^4^ However, partial volume effects, which are caused by the finite thickness of CT slices and the finite size of the target,^5^ are a common issue in MSCT images. This partial volume effect may compromise the reconstruction accuracy of the HDR catheters from the CT images, especially for the catheter tips, if the reconstruction relies on the axial CT images alone. Moreover, the magnitude of the effect can be highly dependent on the type of reconstruction algorithm and kernel used by the commercial CT vendor.^6^, ^7^ Conversely, as a result of a completely different processing scheme, CT scout images have a much higher image resolution in the longitudinal direction, and thus are not affected by the partial volume effect. Previous works, such as that of Yue et. al.,^8^ have demonstrated that by combining orthogonal scout CT images along with the axial images, the radioactive seeds used in low‐dose‐rate (LDR) brachytherapy can be clearly distinguished and the partial source artifact can be reduced in CT‐based brachytherapy planning. The purpose of this work is to further that method into the identification and reconstruction of catheters/needles in HDR brachytherapy treatment and to evaluate whether scout images help to improve the accuracy of the localization especially that of the catheter tips or ends. The accuracies of catheter positions obtained from the developed method were evaluated with a phantom study, as well as with a clinical case. Since both needles and catheters are frequently used in HDR interstitial brachytherapy, both terms will be interchangeably used for the same purpose of description and illustration in the following sections.

## 2 MATERIALS AND METHODS

### 2.A Phantom study

**FIG. 1:**
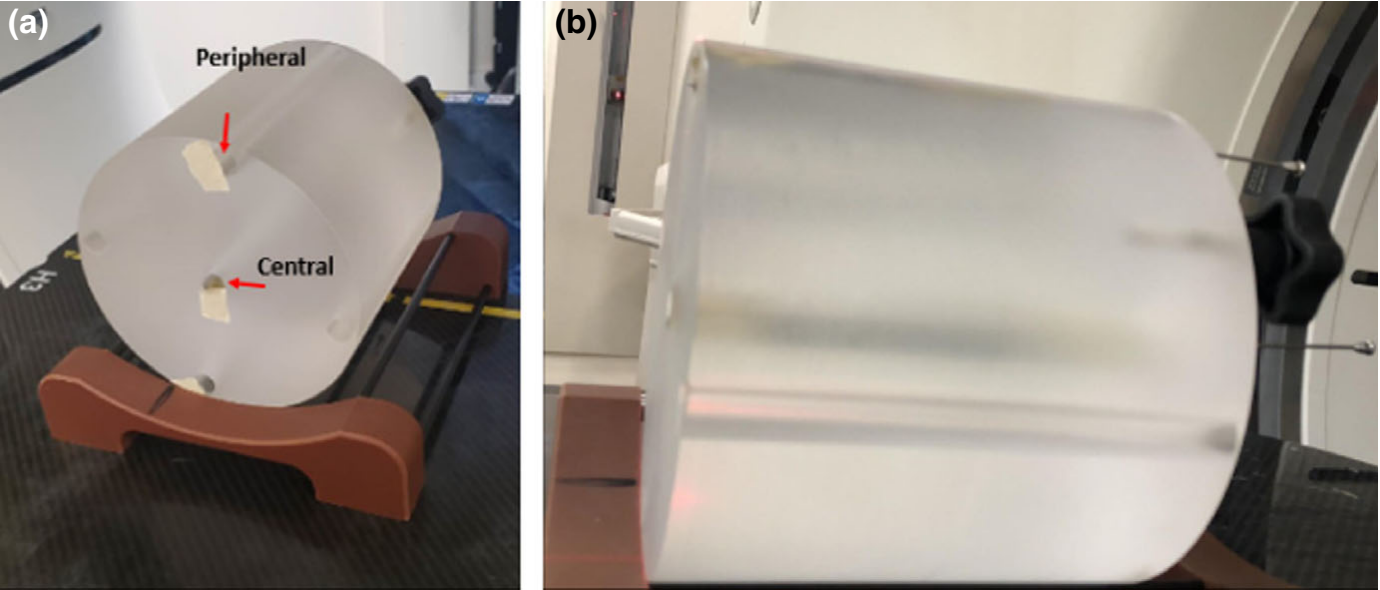
Photo shows the setup for phantom test. Needle was placed in the central hole of the CTDI phantom positioned on a base holder. Then the tip of the needle was aligned to the out bore laser, and sent to the bore for imaging.

In order to best replicate the clinical scenario, a 17‐gauge 25‐cm stainless hollow steel (Alpha Omega Services, Bellflower, CA) needle was inserted into tissue‐equivalent bolus and imaged in a CTDI head phantom (Sun Nuclear, Melbourne, FL) with a GE LightSpeed 16 CT simulator (GE Healthcare, Chicago, IL). A photograph demonstrating the setup of the needles in a CTDI head phantom is shown in Fig. 1. Two plug positions, one in the central and the other in the anterior part of the phantom as a representative of peripheral region, were chosen to investigate the influence of different positions on the localization of needle tips. The lateral part of the phantom was not tested because of the isotropical nature of CTDI phantom in the axial plane. In order to mimic the clinical scenario in which implant needles are immersed in normal human tissues, the stainless needles were inserted into tissue‐equivalent bolus for the scan. Before each CT scan, tips of the needles were aligned to the CT lasers, which were well calibrated. Orthogonal CT scout images and MSCT images were acquired for each of the needle positions. To evaluate the partial volume effect, different slice thicknesses of the helical CT scans were tested, including 0.625 mm, 1.25 mm, 2.5 mm, 5 mm, and 10 mm.

### 2.C Clinical case study

The images of one clinical patient (age 33, female, primary cancer of the cervix) were selected for this study. This patient received brachytherapy boost HDR treatment (30 Gy in 5 fractions) with a Syed interstitial applicator (tandem, cylinder and 16 needles). The treatment was planned based on axial CT images. As part of routine treatment planning, the patient underwent orthogonal CT scout scans and an MSCT scan for pretreatment simulation after needle insertion. The scout scans included an AP scout acquired with 140 kVp and 10 mAs, and a lateral scout (90 degree) with 140 kVp and 40 mAs. The parameters of the CT scan protocol were 1.25 mm slice thickness, 140 kVp, 470 mAs, and 50 cm of FOV.

### 2.D Quantification of the needle positions

The coordinates of a point determined from the scout images and its coordinates determined from the corresponding MSCT images are associated, and the association is briefly described as follows.^8^

On the scout images, in the plane perpendicular to longitudinal direction (head to foot), there is a magnification factor. Assuming the X axis is defined as the horizontal direction (left to right), Y axis is defined as the vertical direction (anterior to posterior), and Z axis is defined as the longitudinal direction (head to foot), the coordinates of a point on the scout image (X_ap, scout_, Y_lat, scout_, Z_scout_) are related to its coordinates on the corresponding CT image (X_CT_, Y_CT_, Z_CT_) as in [Eq. (1)] below.

(1) $$\begin{array}{c} X_{ap,scout}=X_{\mathrm{CT}}\left(\frac{\mathrm{SAD}}{SAD‐Y_{\mathrm{CT}}}\right)\\ X_{lat,scout}=Y_{\mathrm{CT}}\left(\frac{\mathrm{SAD}}{SAD‐X_{\mathrm{CT}}}\right)\\ Z_{\mathrm{scout}}=Z_{\mathrm{CT}} \end{array}$$

SAD is the distance from CT source to the isocenter of the scanner.

The needle tips were identified from scout images and CT images by one experienced medical physicist and independently confirmed by a second physicist. The localization was conducted on a BrachyVision workstation (Varian Medical Systems, Palo Alto, CA) with a Hounsfield unit (HU) window of −1000 to 245 (pelvis window upper limit). For the clinical case, since multiple needles were present, the needle tips were first identified with the MSCT images, then the search was conducted on the scout images near the corresponding tip coordinates calculated based on information from multi‐slice CT images using [Eq. (1)]. The tip was thus identified and localized on the scout images. The purpose of the search was to be able to distinguish clustered needles from each other, and the localized tip coordinates on the scout images were not necessarily identical to the values computed with [Eq. (1)].

**FIG. 2:**
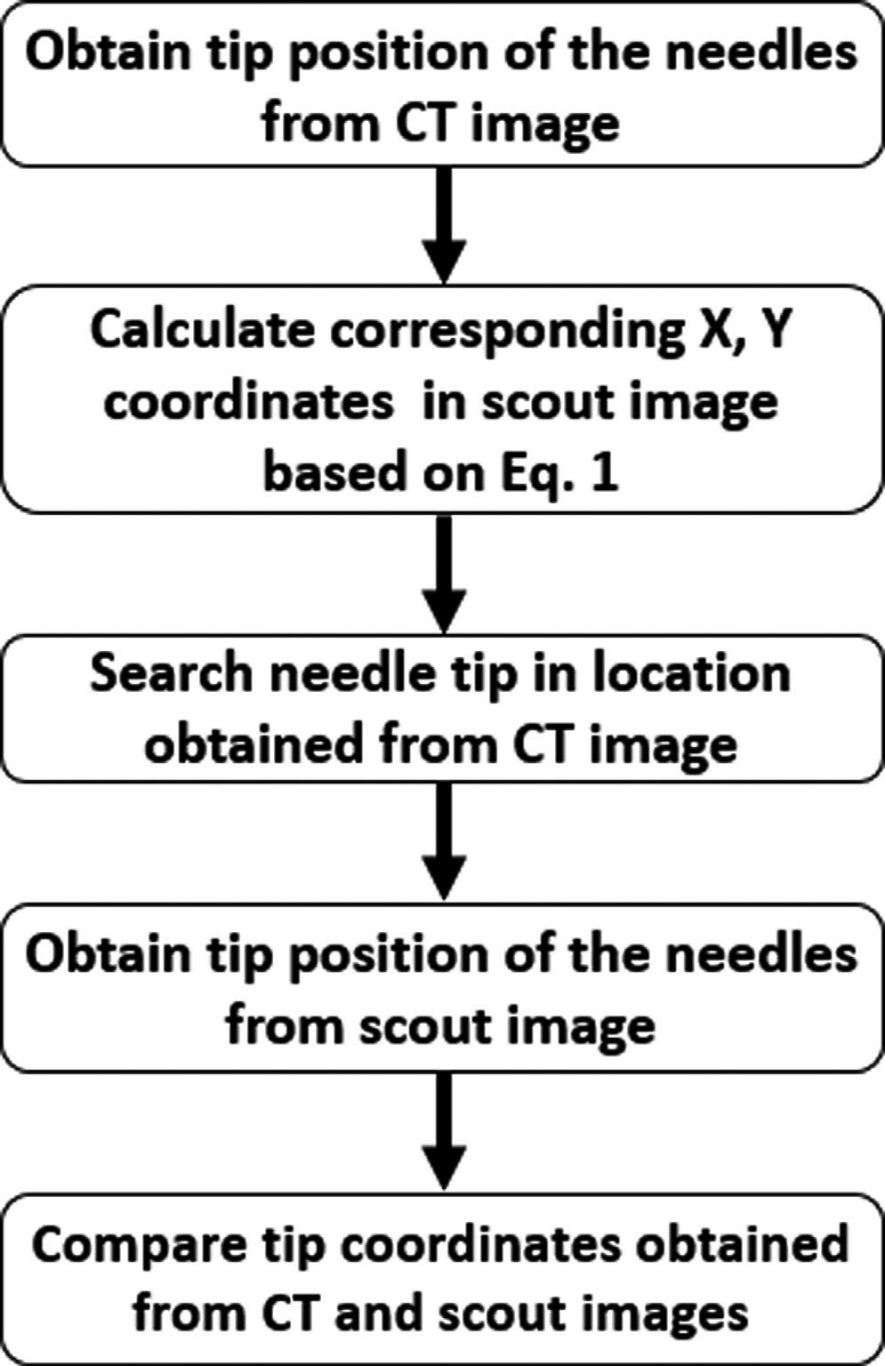
A schematic shows the workflow for quantification of the needle locations in the patient data using scout information.

The coordinates obtained from scout images were then compared quantitatively with the coordinates from the CT image for differences. A detailed schematic of the workflow is shown in Fig. 2.

## 3 RESULTS

### 3.A The Phantom study

**FIG. 3:**
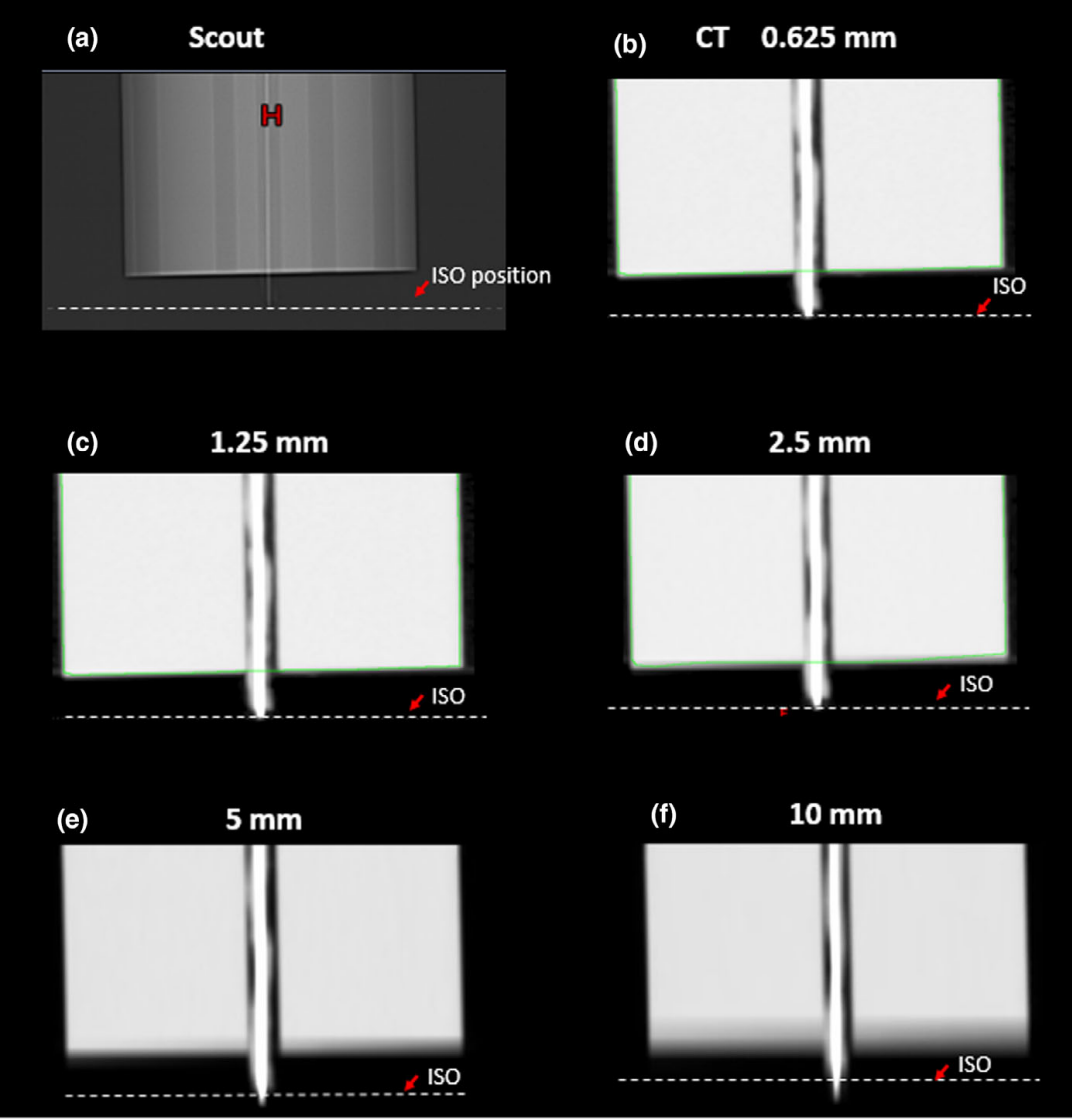
Needle tip shown in a) anterior‐to‐posterior scout image, and b)‐f) CT images with different slice thicknesses. Dash line (ISO) shows the laser position which was aligned to the real needle tip during the setup process. Green lines showed automated contours for body.

Figure 1 shows the setup of a stainless needle placed in the central hole (central plug) of the CTDI head phantom positioned on a base holder. To establish a ground truth for the needle tip position independent of images, the tip of the needle was aligned to the inside laser of the CT system, which was calibrated to be coincident with the imaging isocenter within 1 mm. Thickness of the laser is 1mm at the isocenter. As shown in [Fig. 3(a)], the tip of the needle shown on the scout image was aligned very well with the imaging isocenter position (dashed line) in the longitudinal direction. Thus, the imaging isocenter position could be used as a surrogate reference location of the actual needle tip for the purpose of comparison with the derived needle tip locations from the CT images, with an uncertainty of less than 1 mm. As shown in [Figs. 3(b)‐3(f)], the discrepancy between the derived needle tip location from the CT images and the actual location increased with increasing slice thickness. These data are also listed in Table 1. It is evident that the CT slice thickness could have a significant impact on the accuracy of needle tip localization: the inaccuracy could be as large as 9.6 mm if a 10 mm slice thickness was used for the CT image acquisition and the axial images were used alone for the localization. No significant differences were observed between the scenarios where the needle was respectively placed in the central and the peripheral plugs.

**TABLE 1:** Distance between laser position (real position of needle tips) and tip position shown on CT.

CT resolution	Distance between laser position and tip position on CT (central plug)
0.625 mm	0.8 mm
1.25 mm	1.1 mm
2.5 mm	2.2 mm
5 mm	4.4 mm
10 mm	9.6 mm

### 3.B The Clinical Case Study

**FIG. 4:**
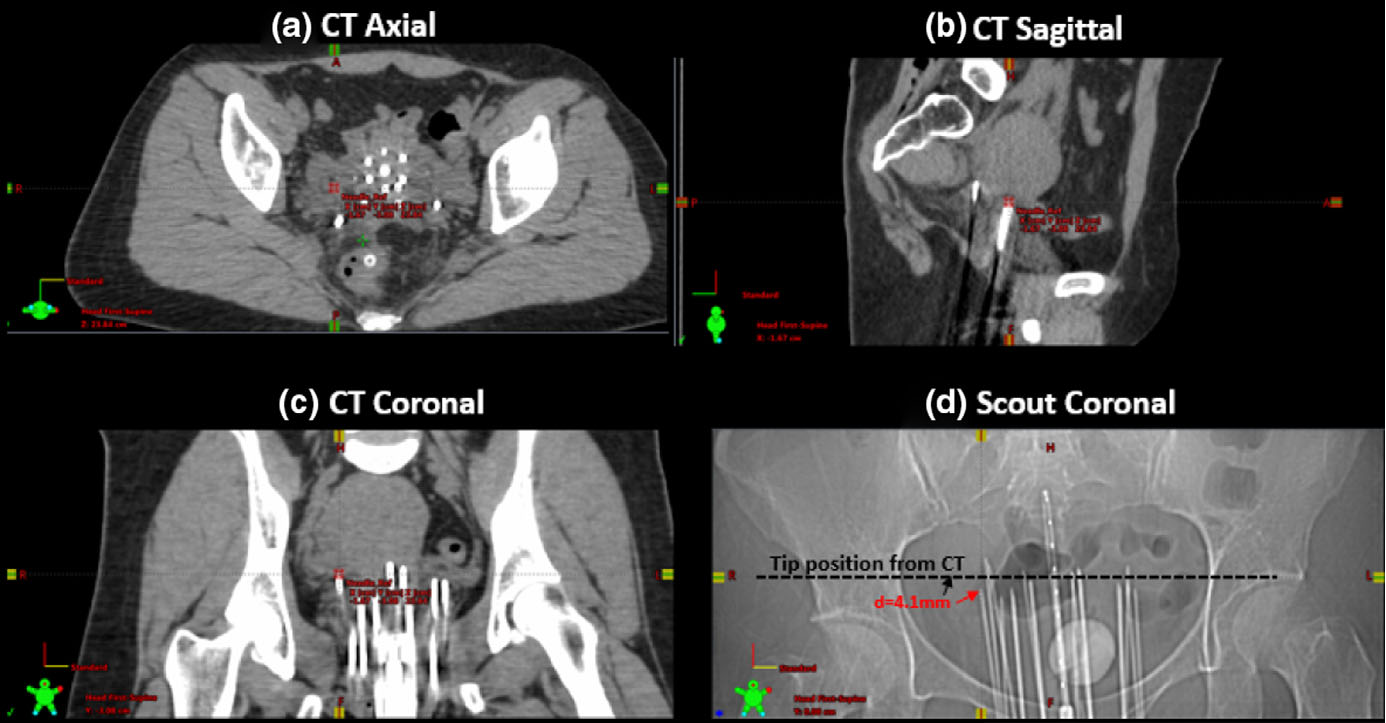
(a)‐(c) Three‐plane MSCT images and (d) an anterior‐to‐posterior scout image from a clinical patient (age 33, female, primary cancer of cervix, treated with interstitial implant for 30 Gy in 5 fractions) show the differences of the needle tip identified from MSCT images and that identified from the scout image.

The needle tip positions were first identified and localized on the MSCT images, as shown in [Fig. 4(a)‐4(c)]. The corresponding coordinates of needle tips in the scout image were derived using [Eq. (1)]. The more accurate tip locations were then searched, identified and localized from the scout image [Fig. 4(d)]. The whole process takes 2–3 min for each needle. In [Fig. 4(d)], the reference positions of the needle tips in the scout image are marked by the black arrow and their counterparts in the axial CT images are shown by the dashed line. The dashed line is located based on the tip location read from CT images. In the worst scenario of this particular case, there was a 4.1 mm difference between the tip location derived using MSCT only and the one using the combination of scout and MSCT images. The mean and standard deviations of the needle tip location differences between the direct identification from CT images and the proposed method combining the scout images were 2.6 mm ± 0.9 mm in the head‐to‐foot direction, 0.4 mm ± 0.2 mm in the left‐to‐right direction, and 0.6 mm ± 0.2 mm in the anterior‐to‐posterior direction, respectively. It is evident that the longitudinal difference was larger than those in the other two directions.

## 4 DISCUSSIONS

In this study, we evaluated the utility of scout images in improving the accuracy of identification and localization of needle tips for HDR brachytherapy treatment. In the phantom study, it was evident that the needle tip location was affected by the CT slice thickness if its determination relied on MSCT alone, especially in the longitudinal direction, while combining the scout images yielded much more reliable results. This finding is not surprising since the spatial resolutions of MSCT are much higher in the lateral and vertical directions on an axial slice (in the submillimeter range) than in the longitudinal direction, along which the spatial resolution is limited by the slice thickness. On the other hand, the scout images allow much higher spatial resolution in the longitudinal direction. Also metallic artifacts caused by the needles are much less on the scout images than on CT images, because the scout imaging acquisition is a 2‐D projection and does not need to undergo filtered back projection or iterative reconstruction. Therefore, adding scout imaging into consideration of digitization can be helpful in more accurately locating the needle positions, especially in the longitudinal (head‐to‐foot) direction. This is particularly important because the tip of needle or catheter serves as an anchor point for treatment planning and dose estimation. Theoretically, if all the tips of needles or catheters are identified with the same distance error, the isodose line will be estimated with identical spatial error by the TPS. In reality, the exact dosimetric impact depends on the magnitude of error, number of needles or catheters and the coverage and volume of CTV for each case. Recently, auto digitization of implanted devices from CT images has been implemented by several vendors, and has been used clinically. The time needed to manually match the tip locations identified from CT and scout images is relatively long (2–3 mins). The target of this work is to implement this approach into the existing automatic digitalization function of commercial brachytherapy TPS software to improve the accuracy and efficiency of automatic digitization. Even manual adjustment is still needed in most scenarios, it will provide a good reference for the planners.

However, there do exist a few potential limitations in using scout images for localization. During interstitial HDR brachytherapy, it is common that multiple needles (>10) are implanted within the patient. The use of this large number of needles or catheters may lead to overlapping on the scout image views and make it more difficult to distinguish one from another on a single image. One possible solution to the problem is adding one or multiple additional oblique scout views. Another potential obstacle is when multiple needles are overlapping along the pathway of the x‐rays in a certain projected scout view, which may lead to significant signal attenuation subsequently causing quantum mottle artifacts^9^ and potentially decreasing the quality of the scout view image.

**FIG. 5:**
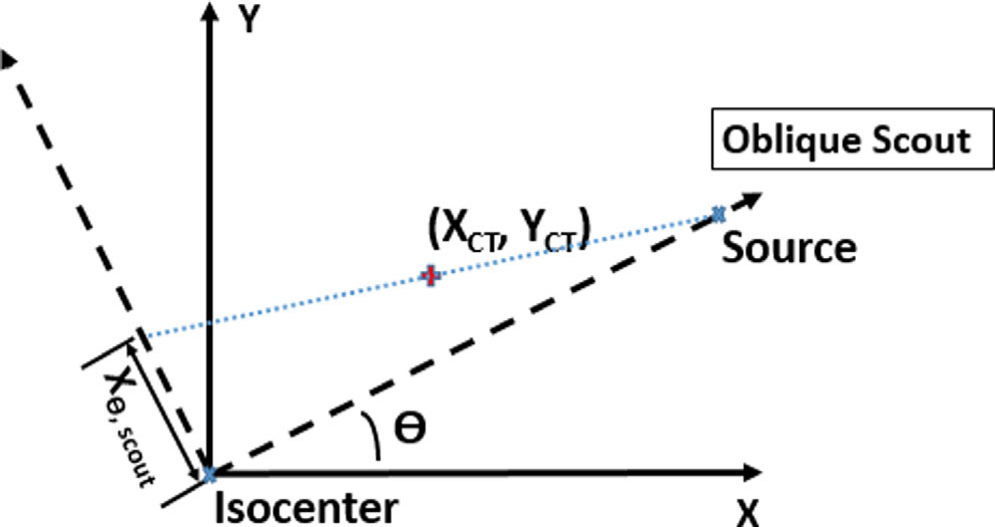
Geometry of projection of a point in space to oblique scout images of CT θ is the angle of the oblique scout from the right lateral scout. X_CT_ and Y _CT_ are the coordinates of the point in CT images, with X in the left‐right direction and Y in the anterior‐posterior direction. X_θ,scout_ is the projection of the point in this oblique scout.

Currently, this proof‐of‐concept study only included one clinical case. And CTDI head phantom used in this study may not be a perfect representation of clinical patient anatomy, due to the variation in size and distance among catheters. Utility and reliability of this method still warrants further investigation in more cases, especially in patients implanted with relatively larger number of needles. Questions such as, how many oblique scout views are required and whether there are some optimal angle combinations still need to be answered. Figure 5 shows a general situation when the oblique scout is θ degree from the right lateral scout (CT gantry angle 90 degree). [Eq. (2)] provides a way to calculate the projection for this arbitrary angle scout. Actually, [Eq. (1)] represents special cases when this θ equals 90, corresponding to AP scout and θ equals 0, corresponding to lateral scout.

(2) $$\begin{array}{c} X_{\theta ,scout}=\left[Y_{\mathrm{CT}}\;\cos\left(\theta \right)‐X_{\mathrm{CT}}\;\sin\left(\theta \right)\right]\left(\frac{\mathrm{SAD}}{SAD‐Y_{\mathrm{CT}}\;\sin\left(\theta \right)‐X_{\mathrm{CT}}\;\cos\left(\theta \right)}\right)\\ Z_{\theta ,scout}=Z_{\mathrm{CT}} \end{array}$$

In conclusion, with this proof‐of‐concept study, we have demonstrated in both phantom and patient studies that the addition of CT scout images together with multi‐slice CT image can improve the accuracy of localization of implanted needle tips, especially in the longitudinal direction. Utilization of CT scout images may potentially improve treatment accuracy in HDR brachytherapy.

## AUTHORS CONTRIBUTIONS

Kun Qing, Ph.D.: Performed the experiment, acquired data, drafted and revised the manuscript. Ning J Yue, Ph.D.: Study design, revision of manuscript. Lara Hathout, M.D.: Provided consultation about clinical background, revised the manuscript. Chi Ma, Ph.D.: Provided consultation about CT imaging acquisition and protocol set up, revised the manuscript. Meral Reyhan, Ph.D.: Revision of manuscript. Jiahua Zhu, Ph.D.: Assisted in data acquisition, revised the manuscript. Ke, Nie, Ph.D.: Revision of manuscript. Gilbert Monte: Assisted in data acquisition and revision of manuscript. Irina Vergalasova, Ph.D.: Study design, data acquisition, revision of manuscript.
